# Association Between Work Status and the Use of Healthcare Services Among Women in the Republic of Korea

**DOI:** 10.1016/j.shaw.2021.10.004

**Published:** 2021-11-01

**Authors:** Min Kyung Hyun, Man-Yee Kan

**Affiliations:** 1 Department of Preventive Medicine, College of Korean Medicine, Dongguk University, Republic of Korea; 2 Department of Sociology, University of Oxford, United Kingdom

**Keywords:** healthcare cost, Korean traditional medicine, patient acceptance of healthcare, woman, work

## Abstract

**Introduction:**

Previous studies on occupational health focussed predominately on the occurrence of occupational diseases. Relatively few studies have measured how employment is associated with the use of healthcare services. This study investigates the association between employment and the extent and range of healthcare use, such as medical expenditures, of women in South Korea.

**Methods:**

We analyze data of the Korean Health Panel, an ongoing longitudinal national representative survey, from 2008 to 2017, to identify the status of economic activity of women by year and age group. We estimate the association between female employment status and medical expenditures by using random effect panel Tobit models. Furthermore, we investigate the association between employment status and the range of healthcare services in biomedicine and traditional Korean medicine (KM) by conducting conditional fixed-effects logistic regression analyses.

**Results:**

For women aged between 25 and 65 in 2017, the majority of them were employed or self-employed. (The proportion of employment of self-employment equals 64.80%). In addition, working women spent 11.6% less on healthcare than nonworking women, and self-employment lowered the healthcare expenditure by 13.1%. Neither work nor the type of work is related to the types and range of healthcare service use. Being employed or self-employed is negatively associated with women’s expenditure on healthcare.

**Conclusions:**

The findings show that employment is associated with less spending on healthcare. They imply that employment has a positive impact on women’s health.

## 1 Introduction

Failing fertility rates and population aging will lead to changes in the labor environment. In 2017, the world average fertility rate was 2.1 children per woman. In addition, cultural restrictions on the participation of women in the labor market are decreasing, and technological advances are improving the productivity of the household, which will affect female employment. Indeed, women and the elderly are making more contributions to the labor demand and supply markets. In particular, the participation of women in the labor force increased from 54% in 1983 to 64% in 2017 [1]. The female employment rate (aged 15 to 64) in the Republic of Korea (ROK) increased from 49.9% in 1990 to 59.4% in 2018, but it was lower than the 79.1% recorded for ROK men in 2018 and below the OECD average of 64.6% [2]. Ironically, the proportion of women with tertiary education among 25–34-year-olds is 75.7%, the highest in the OECD, and 12% higher than 64.1% of ROK men [3].

Many studies have reported negative effects of employment on workers’ health. For example, having a job is associated with a higher chance of developing depression [[4], [5], [6]], pain [[7], [8], [9]], and irregular menstruation [10]. On the other hand, some studies have shown that labor market work is positively associated with health. For example, Kwak and Kim (2018) have found that workers on regular work contracts are healthier than those having temporary jobs.

Across the globe, governments face the challenging problem of increases in healthcare expenditures. The problem is especially serious in the ROK, where the frequency of healthcare utilization is more than double that of the OECD average, and the rate of increase in healthcare expenses has been growing in the last two decades [11,12]. Reducing health expenditure through health promotion and disease prevention is an effective way of achieving a sustainable national health insurance fund [13]. Another way of reducing government healthcare expenditure is to encourage the elderly to participate in some labor market work, e.g. wage work, and part-time work during the day [14].

In this study, we examine the association between female employment and the expenditure on healthcare by analyzing data from the Korean Health Panel Study (KHP). In addition, we investigate if female employment is associated with the concurrent use of biomedicine and traditional Korean medicine (KM).

## 2 Data and variables

### 2.1 Data

Data of this study comes from KHP 2008–2017. The KHP is an ongoing longitudinal survey of a nationally representative Korean population that employed a two-stage cluster method from the population census data of Statistics Korea [15]. The survey has been conducted regularly by the Korea Institute for Health and Social Affairs (KIHSA) and National Health Insurance Corporation (NHIC) to assess the dynamic changes in healthcare utilization, medical expenditure, and health behaviors. Individuals of the sampled households were interviewed annually face-to-face, and the computer-assisted personal interviewing (CAPI) technique was used. The survey questionnaires cover information of both households and individuals, including sociodemographic characteristics, income, living expenses, health level, medical utilization, and medical expenditures.

In this study, we restrict our sample to women aged over 25. The OECD employment statistics are based on individuals aged over 15. We examine women aged over 25 because women in the ROK have the highest rate of tertiary education attainment in the OECD, and therefore, the average age of first employment is late [1,3]. Indeed, according to Statistics Korea, the noneconomic population of housewives and students among women over 15 years of age is approximately 50% of the total female population [16].

### 2.2 Variables selection

What determines whether and how individuals use healthcare services? Current studies have made references to Andersen’s behavioral model of health services utilization and Grossman’s health demand model [17,18]. Andersen’s model suggests that the utilization of health services is affected by three main factors: (1) predisposing factors determined by sociodemographic characteristics of individuals such as age and educational qualifications that exist before their illness; (2) enabling factors determined by resources and knowledge how to access to healthcare services such as economic income and insurance; and (3) need factors that are determined by people perception of their general health and diseases [19]. Grossman (1972), on the other hand, constructed a health demand model for the commodity of “good health,” which helps explain individuals’ variations in health-related behavior and healthcare utilization [17]. Grossman proposed that individuals consume “health” itself rather than the healthcare services when they use the healthcare services. In other words, individuals invest in their health by using healthcare services so as maximize healthy time for work and leisure [20]. Therefore, the level of public healthcare services will influence the amount and productivity of labor supplied to an economy. From users’ perspective, what consumers demand when they purchase medical services are not these services per se but rather “good health” [21].

In this study, the variables related to social and economic factors and health factors were selected based on Andersen’s behavioral model of health services utilization and Grossman’s health demand model. The variables related to social and economic factors include age, marital status, education period, household income, types of health insurance, and types of pension (national & private). In addition, the variables related to health factors include the frequencies of using outpatient medical service, over-the-counter (OTC) drug use in the last three months, and physical activities.

### 2.3 Dependent variables

#### 2.3.1 Medical expenditure

Our main dependent variable is the medical expenditure, which is defined as the sum of expenses on emergency medical care services, emergency prescription drugs, emergency transportation (ambulance), hospitalization, care charges for inpatients, inpatient prescription drugs, outpatient medical services, outpatient prescription drugs, and hospital travels.

#### 2.3.2 Types of healthcare services utilization

Another dependent variable is whether biomedicine and KM were used concurrently. The Republic of Korea legally allows the use of KM as a traditional medicine along with biomedicine, and patients use KM according to their preference. A previous study reported that the utilization rate was high in women [22]. Concurrent users are defined as respondents who used both biomedicine and KM in the last year, and biomedicine users are defined as respondents who used biomedicine only in the last year.

### 2.4 Key independent variable

#### 2.4.1 Employment status

We use two key independent variables related to work and employment. First, it is whether or not the individual is engaged in economic activity during the survey period. Second is the employment status measured in four categories: no work, unpaid family work, self-employed work, and wage work.

### 2.5 Control variables

#### 2.5.1 Household income

Household income refers to household equalized income calculated by dividing the total household income by the square root of the number of household members based on the square root scale.

#### 2.5.2 Types of health insurance

The Korean national healthcare system covers the entire population residing within the territory of the ROK except for beneficiaries of medical aid (who are excluded from the coverage of the national health insurance according to the National Health Insurance Act). Approximately 97% of Koreans have national health insurance, which is divided into two groups: employee insured and self-employed insured. The employee insured includes workers and employers in all workplaces, public officials, private school employees, and daily-paid workers at construction sites. Premiums are paid by the employer and the employee on a 50:50 basis. The self-employed insured includes farmers, fishermen, and self-employed persons. Their contribution amount depends on their income, properties, living standard, and level of participation in economic activities.

#### 2.5.3 Physical activity

The KHP measures the intensity of physical activity using the International Physical Activity Questionnaire (IPAQ) short form. The items in the IPAQ short form were structured to provide separate scores on walking, moderate-intensity, and vigorous-intensity activity. Calculation of the total score for each type of physical activity requires multiplication of the duration (minutes) and frequency per week (days) and the weight of each type of physical activity. The weightage for walking, moderate intensity, and vigorous intensity was 3.3, 4.0, and 8.0, respectively. The total physical activity is the sum of walking, moderate-intensity, and vigorous-intensity activity. The categorical variables were classified into three categories (low, moderate, and high) according to their classification method using the IPAQ scoring protocol [23].

## 3 Statistical analysis

In what follows, we first present some descriptive statistics about the economic activities of women. We then estimate the association between employment status and medical expenditures of women by conducting panel Tobit regression models, which are also known as censored regression models. This is because medical expenditures are left-censored data with a value of zero, and the medical expenditure data have been log-transformed to give a log-normal distribution. We have conducted a likelihood-ratio test that formally compares the pooled Tobit model estimation with the random-effect-panel Tobit model estimation, and the results confirm that the random-effects Tobit model is an appropriate method. The estimation results such as coefficients and standard errors are calculated based on the random-effects Tobit model of all data, including left-censored outcomes. As interpreting the coefficients of the nonlinear model is difficult, we also present the marginal effect, which is estimated based on the actual outcome values and the effect estimates between covariates. The marginal effect estimation coefficients are calculated by differentiating the random-effects model with respect to the covariates. We further conduct conditional fixed-effects logistic regression models to estimate how employment status is associated with the types of medical services used. Conditional fixed-effects logistic regression models can be used for panel data in which the same subject is measured at more than one time point. The Hausman specification test is applied to select between fixed and random effects models [24]. The null hypothesis is that there is no correlation between the unique errors and the regressors in the model, and the null hypothesis is that the preferred model is random effects and the alternative fixed model. A two-sided p-value of <0.001, <0.01, or <0.05 is considered significant. All data manipulation and statistical analyses were performed using Stata/MP version 16 (StataCorp LP, College Station, TX, USA).

## 4 Results

### 4.1 Economic activities of women by year

**Fig. 1:**
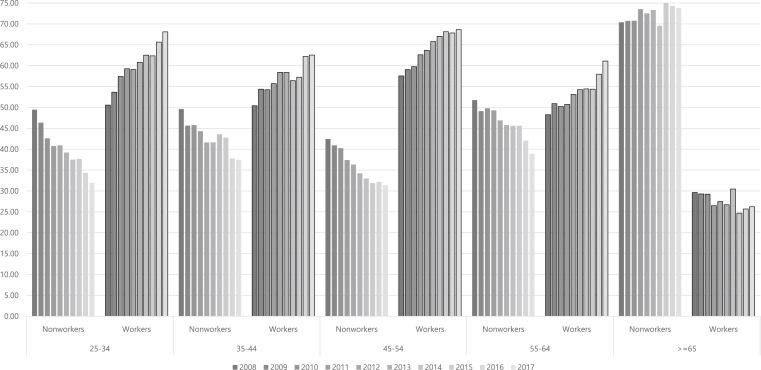
Economic activity of women according to the year.

The proportion of workers among women over the age of 25 years increased from 47.18% in 2008 to 51.16% in 2017. In contrast, the proportion of workers from those 25 to 64 years old increased from 51.81% in 2008 to 64.80% in 2017. In other words, the proportion of workers excluding those over 65 was greater than nonworkers at all ages (Fig. 1).

**Fig. 2:**
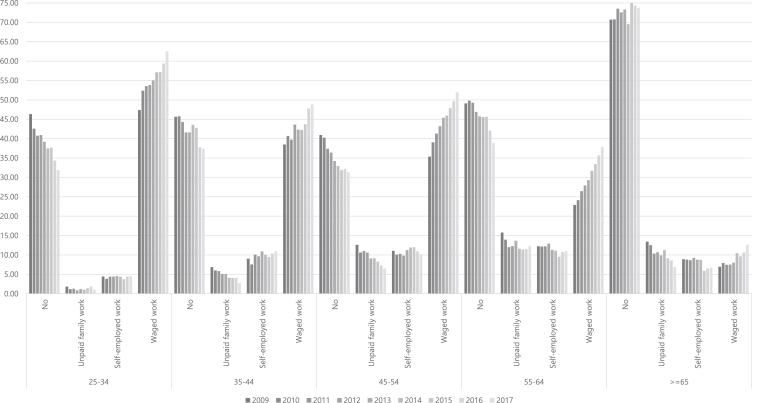
Work types of women according to the year.

The proportion of wage workers was higher than unpaid family workers and self-employed workers, and the proportion of wage workers in the 25–34 year age group was the highest with 47–63% in total, less than 28% in the 55–64 age group, and less than 13% in the 65 years old group (Fig. 2).

### 4.2 Association between women’s employment and medical expenditure

From Table 1, we can see that working women spent less on medical care than nonworking women. The following women had less medical expenditures in the 25–34-year-old group: those who have employee insurance and those who performed low-intensity physical activities. In the 35–45-year-old group, women who were on self-employed insurance or medical aid insurance, and those who perform moderate or high-intensity physical activities had less medical expenditures than other women. On the other hand, in the 25–34-year-old group, those who were single, had six years or less of education, had the lowest household equalized income, did not have any pension, used lesser outpatient medical services, and had not taken OTC drugs, spent more on medical care. For women older than 45 years, those who were married, had more than seven years of education, had higher household equalized income, had pensions, used outpatient medical service more frequently, and had taken OTC drugs in the last three months spent more on medical care. Results of the marginal effects show that the working women spent 11.6% less on medical care than nonworking women. In addition, the 35–44-year-old group spent 10.4% less on medical care than the 25–34-year-old group, and the high-intensity physical activity group spent 13.9% less on medical care than the low-intensity physical activity group (Table 1).

**Table 1:** Women’s employment status and medical expenditure

		Work						Work types					
		Estimate result			Marginal effect			Estimate result			Marginal effect		
		Coef.	95% CI		Coef.	95% CI		Coef.	95% CI		Coef.	95% CI	
Have work	No	Ref.			Ref.								
	Yes	–0.116∗∗∗	–0.146	–0.086	–0.116∗∗∗	–0.146	–0.086						
Work types	No							Ref.			Ref.		
	Unpaid family work							–0.116∗∗∗	–0.172	–0.061	–0.116∗∗∗	–0.172	–0.061
	Self-employed work							–0.131∗∗∗	–0.183	–0.080	–0.131∗∗∗	–0.183	–0.080
	Waged work							–0.112∗∗∗	–0.146	–0.077	–0.112∗∗∗	–0.146	–0.077
**Social and Economic Factors**													
Age group	25–34	Ref.			Ref.			Ref.			Ref.		
	35–44	–0.104∗∗∗	–0.160	–0.049	–0.104∗∗∗	–0.160	–0.049	–0.103∗∗∗	–0.159	–0.048	–0.103∗∗∗	–0.159	–0.048
	45–54	0.216∗∗∗	0.154	0.278	0.216∗∗∗	0.154	0.278	0.217∗∗∗	0.155	0.280	0.217∗∗∗	0.155	0.280
	55–64	0.524∗∗∗	0.456	0.592	0.524∗∗∗	0.456	0.592	0.525∗∗∗	0.457	0.593	0.525∗∗∗	0.457	0.593
	≥65	0.575∗∗∗	0.501	0.649	0.575∗∗∗	0.501	0.649	0.577∗∗∗	0.503	0.650	0.577∗∗∗	0.503	0.650
Marital status	Single	Ref.			Ref.			Ref.			Ref.		
	Married	0.157∗∗∗	0.116	0.199	0.157∗∗∗	0.116	0.199	0.158∗∗∗	0.115	0.200	0.158∗∗∗	0.115	0.200
Education (years)	≤6	Ref.			Ref.			Ref.			Ref.		
	7–12	0.064∗	0.009	0.119	0.064∗	0.009	0.119	0.063∗	0.008	0.118	0.063∗	0.008	0.118
	13≤	0.012	–0.058	0.082	0.012	–0.058	0.082	0.012	–0.059	0.082	0.012	–0.059	0.082
Household equivalized income (Quintile)	First (lowest)	Ref.			Ref.			Ref.			Ref.		
	Second	0.066∗∗	0.023	0.108	0.066∗∗	0.023	0.108	0.065∗∗	0.023	0.108	0.065∗∗	0.023	0.108
	Third	0.108∗∗∗	0.061	0.156	0.108∗∗∗	0.061	0.156	0.109∗∗∗	0.061	0.156	0.109∗∗∗	0.061	0.156
	Fourth	0.182∗∗∗	0.132	0.232	0.182∗∗∗	0.132	0.232	0.182∗∗∗	0.132	0.232	0.182∗∗∗	0.132	0.232
	Fifth (highest)	0.251∗∗∗	0.197	0.304	0.251∗∗∗	0.197	0.304	0.251∗∗∗	0.198	0.304	0.251∗∗∗	0.198	0.304
Type of health insurance	Employee	Ref.			Ref.			Ref.			Ref.		
	Self-employed	–0.065∗∗∗	–0.097	–0.032	–0.065∗∗∗	–0.097	–0.032	–0.063∗∗∗	–0.096	–0.030	–0.063∗∗∗	–0.096	–0.030
	Medical aid	–1.046∗∗∗	–1.122	–0.970	–1.046∗∗∗	–1.122	–0.970	–1.046∗∗∗	–1.122	-0.970	–1.046∗∗∗	–1.122	–0.970
Pension (national or private)	No	Ref.			Ref.			Ref.			Ref.		
	Yes	0.116∗∗∗	0.085	0.146	0.116∗∗∗	0.085	0.146	0.115∗∗∗	0.084	0.146	0.115∗∗∗	0.084	0.146
**Health Factors**													
Frequency of outpatient medical service		0.027∗∗∗	0.026	0.027	0.027∗∗∗	0.026	0.027	0.027∗∗∗	0.026	0.027	0.027∗∗∗	0.026	0.027
OTC drug use for three months	No	Ref.			Ref.			Ref.			Ref.		
	Yes	0.071∗∗∗	0.038	0.105	0.071∗∗∗	0.038	0.105	0.071∗∗∗	0.037	0.105	0.071∗∗∗	0.037	0.105
Physical activity *(Missing 2008)*	Low	Ref.			Ref.			Ref.			Ref.		
	Moderate	–0.068∗∗∗	–0.092	–0.043	–0.068∗∗∗	–0.092	–0.043	–0.068∗∗∗	–0.092	-0.043	–0.068∗∗∗	–0.092	–0.043
	High	–0.139∗∗∗	–0.184	–0.094	–0.139∗∗∗	–0.184	–0.094	–0.139	–0.184	-0.094	–0.139∗∗∗	–0.184	–0.094
Constant		11.488∗∗∗	11.402	11.574				11.486∗∗∗	11.400	11.573			
/sigma_u		0.756∗∗∗	0.738	0.773				0.756∗∗∗	0.739	0.774			
/sigma_e		1.135∗∗∗	1.127	1.143				1.135∗∗∗	1.126	1.143			
rho		0.307	0.297	0.318				0.307	0.297	0.318			

∗∗∗*p* < 0.001, ∗∗*p* < 0.01, ∗*p* < 0.05.

Abbreviations: CI, Confidence Intervals; Coef, Coefficient.

Working women, regardless of work types, i.e. employed work, unpaid family work, self-employed work, and wage work, had less medical expenditure than nonworking women. The positive or negative effects of all variables included in this analysis on medical expenditures were the same as in the above analysis. Results of the marginal effects indicate that unpaid family workers spent 11.6% less on medical care than nonworking women. Moreover, those who did self-employed work and wage work spent 13.1% and 11.2% less on medical care than nonworking women (Table 1).

### 4.3 Types of healthcare services

As can be seen in Table 2, the employment status of women is not associated significantly with the likelihood of concurrent use of biomedicine and KM. However, concurrent users of biomedicine and KM had a higher frequency of using outpatient medical services than those who used biomedicine only (OR: 1.053, 95% CI: 1.050–1.055) (Table 2).

**Table 2:** Factors associated with the concurrent use of biomedicine and KM by women

		Work			Work types		
		OR	95% CI		OR	95% CI	
Work	No	Ref.					
	Yes	1.039	0.945	1.144			
Work types	No				Ref.		
	Unpaid family work				0.988	0.819	1.190
	Self-employed work				1.023	0.870	1.204
	Waged work				1.056	0.947	1.177
**Social and Economic Factors**							
Age group	25–34	Ref.			Ref.		
	35–44	1.074	0.858	1.344	1.075	0.859	1.345
	45–54	1.231	0.912	1.660	1.232	0.913	1.662
	55–64	1.187	0.824	1.709	1.189	0.825	1.713
	≥65	1.252	0.812	1.931	1.254	0.813	1.933
Marital status	Single	Ref.			Ref.		
	Married	0.877	0.712	1.081	0.882	0.715	1.087
Education (years)	≤6	Ref.			Ref.		
	7–12	0.866	0.530	1.413	0.863	0.529	1.408
	13≤	0.871	0.394	1.925	0.870	0.394	1.923
Household equivalized income (Quintile)	First (lowest)	Ref.			Ref.		
	Second	0.889	0.783	1.011	0.890	0.783	1.011
	Third	0.959	0.828	1.112	0.959	0.828	1.112
	Fourth	0.981	0.835	1.152	0.980	0.835	1.151
	Fifth (highest)	0.913	0.765	1.090	0.912	0.764	1.088
Type of health insurance	Employee	Ref.			Ref.		
	Self-employed	0.972	0.873	1.082	0.976	0.876	1.087
	Medical aid	0.826	0.595	1.147	0.830	0.598	1.152
Pension (national or private)	No	Ref.			Ref.		
	Yes	1.107∗	1.003	1.223	1.104	1.000	1.220
**Health Factors**							
Frequency of outpatient medical service		1.053∗∗∗	1.050	1.055	1.053∗∗∗	1.050	1.055
OTC drug use for three months	No	Ref.			Ref.		
	Yes	1.060	0.970	1.160	1.061	0.970	1.160
Physical activity (Missing 2008)	Low	Ref.			Ref.		
	Moderate	1.020	0.954	1.091	1.020	0.954	1.091
	High	0.966	0.854	1.092	0.967	0.856	1.094
Year	2010	Ref.			Ref.		
	2011	0.859∗	0.766	0.965	0.859∗	0.765	0.964
	2012	0.914	0.812	1.029	0.912	0.810	1.027
	2013	0.946	0.837	1.069	0.945	0.836	1.068
	2014	0.837∗∗	0.740	0.945	0.836∗∗	0.739	0.944
	2015	0.833∗∗	0.734	0.946	0.831∗∗	0.732	0.944
	2016	0.773∗∗∗	0.678	0.882	0.772∗∗∗	0.676	0.881
	2017	0.843∗	0.736	0.965	0.841∗	0.734	0.963

∗∗∗*p* < 0.001, ∗∗*p* < 0.01, ∗*p* < 0.05.

Abbreviations: CI, Confidence interval; OR, Odds Ratio.

## 5 Discussion and conclusion

More than half of women aged 25 to 64 are working, with the highest proportion noted in 2017 (64.8%). The female employment rate exceeded 50% in Statistics Korea and OECD reports; 50.7% of women aged 15 and over in 2017 were employed, and the female employment rate of women aged between 15 and 64 was 59.4% in 2018 [2,16]. This is lower than the figures in Germany, the UK, and the USA (74.3%, 73.6%, and 68.2% respectively) and much lower than that in Japan (71.3%) [2,24].

There were more wage workers than unpaid family work and self-employed work in all age groups, except women aged 65 or older. On the other hand, the proportion of wage workers was the highest in the 25 to 34 years old group, and the proportion decreased with age. This is because many women reduced their labor market participation after marriage and childbirth. In ROK, the average age of marriage and childbirth is over 30 years old, which is the highest among OECD countries, and the rate of career breaks due to marriage, childbirth, and parenting was more than 20% in 2017 [25,26]. Gender discrimination against married women still exists in the labor market in the ROK [27,28].

On the other hand, young women are more likely to be employed in wage work than older women because of the increases in educational qualifications across cohorts, which facilitate labor market participation [29]. The proportion of 55–64-year-old women with tertiary educational attainment was (15.5%), compared with 75.7% for those aged 25–34 [3].

Women workers face many challenges such as discrimination in pay, career opportunities, and job, and making balances between work and family [[30], [31], [32]]. Although some earlier studies reported positive relationships between work and health, as mentioned in the introduction, employment was considered to be the main factor of diseases [6,8,9,[33], [34], [35]]. Various scholars have suggested policy interventions to promote workplace health [[36], [37], [38], [39]] and those to help workers return to work after illness [[40], [41], [42]]. These studies all suggest that work can negatively affect health. Nevertheless, after people return to work after illness, current labor policies focus on helping them to restore their daily lives rather than helping them to regain their ability to perform work. This might be because work is often regarded only as a means of economic and social welfare, and wages are also considered as compensation for the health loss [43].

This study has investigated the relationship between employment status and medical expenditures. Female employment is associated with lesser medical expenditures. Urtasun also suggested that work can have a positive effect on health when the working conditions are good, and efforts are below a moderate effort threshold [43]. The benefits of employment apply to both employed and self-employed women. Our findings show that self-employed women spent less on medical care than nonworking women. However, we did not find evidence of the dominant effect of wage work compared to a previous study on the elderly [14,44]. This is likely because self-employed work is beneficial to women who are responsible for childcare and suffer from pressures in work-life balance [44].

Employment may have positive impacts on health through the following mechanisms. First, employment acts as a main source of financial income for most of the people. Financial resources gained through employment provide access to food, goods, and services that are beneficial for health and a healthy lifestyle [45,46]. Second, through employment, individuals are provided with pensions, health insurance schemes, and other benefits, which can have positive impacts on health [47,48]. Furthermore, studies show that employment can enhance psychological well-being [49,50].

The continued increase in healthcare costs is a global problem. Studies have been conducted to investigate factors associated with lower healthcare costs. According to a report from the American Medical Association, the five factors that affect the cost of healthcare are population growth, aging population, disease prevalence or incidence, medical service utilization, and service price and intensity [51,52]. For example, among the European Union countries, aging is a major factor in healthcare spending per person [53]. In this study, we have found that age and medical service utilization, such as outpatient medical service use frequency and OTC drug use, are positively associated with medical expenditure. In addition, there have been reports that physical activities are related to a reduction in healthcare expenditure [54]. In the present study, a higher intensity of physical activities is associated with a lower level of medical expenditure.

Many studies have reported that women are more likely to use complementary therapies alongside biomedicine than men [22,55]. On the other hand, few studies have explained the variations among women. In this study, we have found that the frequency of outpatient use is associated with a higher chance of concurrent use of biomedicine and KM. In other words, there is no evidence to show that employment status or types of employment are associated with the chance of concurrent use of biomedicine and KM.

This study had some limitations. First, some important variables are not available from the current data source. The impact of long or night shift work on health could not be analyzed in the present study. Existing studies have found that long or night shifts work is a risk factor for morbidities, such as obesity, fertility, and multiple sclerosis [[56], [57], [58]]. [43] have shown that long or night shift work is more likely to have a negative health effect. Further research should look into the effects of work conditions on health.

In addition, although we control for marital status in the regression models, it was not possible to take account of family responsibilities such as housework and childcare. Finally, medical expenditure was used as a surrogate outcome for health in this study. Although it has been used extensively in medical research, there are limitations to this indicator. Further studies can focus on specific disease outcomes in order to estimate precisely the effect of employment health [59,60].

Finally, we used conditional fixed-effects logistic regression models to control for the time-constant characteristics of the respondents. However, it is possible that the sample might have systematically missed less healthy respondents. The “healthy worker effect” might not have been completed controlled in our models.

In sum, we have found that all types of female employment are associated with a reduction in medical expenditure. Policymakers should consider promoting female employment as a means to improve women’s health and reduce public expenditure on medical services.

## Funding source

This study was supported by the 10.13039/501100003725National Research Foundation of Korea (NRF) grant funded by the Korea government (Ministry of Science and ICT) (No. 2016R1C1B3006806), and an 10.13039/501100000781European Research Council Consolidator Grant Award (awardee: Man-Yee Kan, grant number 771736).

## Conflicts of interest

The authors have no conflicts of interest to declare with respect to the authorship and/or publication of this article.

## References

[1] OECD. OECD Employment Outlook 2019: The Future of Work. 10.1787/9ee00155-en2019.

[2] OECD. OECD Labour Force Statistics 2019. 10.1787/g2g9fb3e-en2019.

[3] OECD. Education at a Glance 2019: OECD Indicators. 10.1787/f8d7880d-en2019.

[4] Hoshino A., Amano S., Suzuki K., Suwa M. (2016). Relationships between depression and stress factors in housework and paid work among Japanese women. Hong Kong J Occup Ther.

[5] Leupp K. (2017). Depression, work and family roles, and the gendered life course. J Health Soc Behav.

[6] Kim S.Y., Shin Y.C., Oh K.S., Shin D.W., Lim W.J., Cho S.J. (2019). Gender and age differences in the association between work stress and incident depressive symptoms among Korean employees: a cohort study. Int Arch Occup Environ Health.

[7] Fjell Y., Alexanderson K., Nordenmark M., Bildt C. (2008). Perceived physical strain in paid and unpaid work and the work-home interface: the associations with musculoskeletal pain and fatigue among public employees. Women Health.

[8] Mamunur R., Marja-Leena K., Marina H., Annika N. (2018). Factors related to work ability and well-being among women on sick leave due to long-term pain in the neck/shoulders and/or back: a cross-sectional study. BMC Public Health.

[9] Grant K.M.K., Vo T., Tiong L.U. (2019). The painful truth: work-related musculoskeletal disorders in Australian surgeons. Occup Med (Oxford, England).

[10] Kwak Y., Kim Y. (2018). Irregular menstruation according to occupational status. Women Health.

[11] OECD. Health at a Glance 2019: OECD Indicators. 10.1787/4dd50c09-en2019.

[12] McKee M. (2018). Global sustainable healthcare. Medicine.

[13] Sperlich S., Geyer S. (2015). The mediating effect of effort-reward imbalance in household and family work on the relationship between education and women's health. Soc Sci Med.

[14] Hyun M.K. (2018). Effect of work on medical expenditures by elderly: findings from the Korean health panel 2008–2013. Saf Health Work.

[15] KHP. The Korea Health Panel Survey. https://www.khp.re.kr:444/eng/survey/sampling.do2019.

[16] KOSIS (2020). http://kosis.kr/statHtml/statHtml.do?orgId=101&tblId=DT_1DA7001.

[17] Grossman M. (1972). On the concept of health capital and the demand for health. J Polit Econ.

[18] Andersen R., Newman J.F. (1973). Societal and individual determinants of medical care utilization in the United States. Milbank Mem Fund Q Health Soc.

[19] Andersen R.M. (1995). Revisiting the behavioral model and access to medical care: does it matter?. J Health Soc Behav.

[20] Grossman M. (2000). Handbook of health economics.

[21] Jacobson L. (2000). The family as producer of health — an extended grossman model. J Health Econ.

[22] Hyun M.K. (2019). Determinants of the concurrent use of biomedicine and Korean Medicine: a study based on the Korean Health Panel survey (2008–2014). Eur J Integr Med.

[23] IPAQ. Guidelines for Data Processing and Analysis of the International Physical Activity Questionnaire (IPAQ) – Short and Long Forms. https://sites.google.com/site/theipaq/2005.

[24] Takeuchi M., Tsutsui J. (2016). Combining egalitarian working lives with traditional attitudes: gender role attitudes in Taiwan, Japan, and Korea. Int J Japanese Sociol.

[25] OECD (2019). http://www.oecd.org/els/family/database.htm.

[26] KOSTAT (2019). https://index.go.kr/potal/visual/VisualDtlPageDetail.do?idx_cd=3038.

[27] Lee Y-s, Eun K.-S. (2005). Attitudes toward married women’s employment in Korea and Japan: implications from latent class analyses. Dev Soc.

[28] Lee B.S., Jang S., Sarkar J. (2008). Women's labor force participation and marriage: the case of Korea. J Asian Econ.

[29] Jung J., Lee S.J. (2016). Influence of university prestige on graduate wage and job satisfaction: the case of South Korea. J High Educ Policy Manag.

[30] Hakim C. (2004).

[31] Echeverri-Carroll E.L., Oden M.D., Gibson D.V., Johnston E.A. (2018). Unintended consequences on gender diversity of high-tech growth and labor market polarization. Res Policy.

[32] Critoph U. (2006).

[33] Virtanen M., Jokela M., Madsen I., Hanson L.L.M., Lallukka T., Nyberg S. (2018). Long working hours and depressive symptoms: systematic review and meta-analysis of published studies and unpublished individual participant data. Scand J Work Environ Health.

[34] Harvey S.B., Modini M., Joyce S., Milligan-Saville J.S., Tan L., Mykletun A. (2017). Can work make you mentally ill? A systematic meta-review of work-related risk factors for common mental health problems. Occup Environ Med.

[35] Duchaine C.S., Aube K., Gilbert-Ouimet M., Vezina M., Ndjaboue R., Massamba V. (2020). Psychosocial stressors at work and the risk of sickness absence due to a diagnosed mental disorder: a systematic review and meta-analysis. JAMA Psychiatry.

[36] Sundstrup E., Seeberg K.G.V., Bengtsen E., Andersen L.L. (2020). A systematic review of workplace interventions to rehabilitate musculoskeletal disorders among employees with physical demanding work. J Occup Rehabil.

[37] van de Ven D., Robroek S.J.W., Burdorf A. (2020). Are workplace health promotion programmes effective for all socioeconomic groups? A systematic review. Occup Environ Med.

[38] Tarro L., Llaurado E., Ulldemolins G., Hermoso P., Sola R. (2020). Effectiveness of workplace interventions for improving absenteeism, productivity, and work ability of employees: a systematic review and meta-analysis of randomized controlled trials. Int J Environ Res Public Health.

[39] Stanulewicz N., Knox E., Narayanasamy M., Shivji N., Khunti K., Blake H. (2019). Effectiveness of lifestyle health promotion interventions for nurses: a systematic review. Int J Environ Res Public Health.

[40] Etuknwa A., Daniels K., Eib C. (2019). Sustainable return to work: a systematic review focusing on personal and social factors. J Occup Rehabil.

[41] Westerlind E., Persson H.C., Eriksson M., Norrving B., Sunnerhagen K.S. (2020). Return to work after stroke: a Swedish nationwide registry-based study. Acta Neurol Scand.

[42] Endo M., Haruyama Y., Muto G., Yokoyama K., Kojimahara N., Yamaguchi N. (2018). Employment sustainability after return to work among Japanese stroke survivors. Int Arch Occup Environ Health.

[43] Urtasun A., Nunez I. (2018). Healthy working days: the (positive) effect of work effort on occupational health from a human capital approach. Soc Sci Med.

[44] Damaske S., Frech A. (2016). Women's work pathways across the life course. Demography.

[45] Ettner S.L. (1996). New evidence on the relationship between income and health. J Health Econ.

[46] Ecob R., Davey Smith G. (1999). Income and health: what is the nature of the relationship?. Soc Sci Med.

[47] Cheng L., Liu H., Zhang Y., Zhao Z. (2018). The health implications of social pensions: evidence from China's new rural pension scheme. J Comp Econ.

[48] Pak T.-Y. (2021). What are the effects of expanding social pension on health? Evidence from the Basic Pension in South Korea. J Econ Ageing.

[49] Winefield A.H., Tiggemann M. (1990). Employment status and psychological well-being: a longitudinal study. J Appl Psychol.

[50] Warr P., Parry G. (1982). Paid employment and women's psychological well-being. Psychol Bull.

[51] Rama A. (2019).

[52] Dieleman J.L., Squires E., Bui A.L., Campbell M., Chapin A., Hamavid H. (2017). Factors associated with increases in US health care spending, 1996-2013. Jama.

[53] Williams G.A., Cylus J., Roubal T., Ong P., Barber S., Sagan A., Normand C., Figueras J., North J., White C. (2019). Sustainable health financing with an ageing population: will population ageing lead to uncontrolled health expenditure growth? Copenhagen (Denmark): European Observatory on Health Systems and Policies.

[54] Andreyeva T., Sturm R. (2006). Physical activity and changes in health care costs in late middle age. J Phys Act Health.

[55] Kaur J., Hamajima N., Yamamoto E., Saw Y.M., Kariya T., Soon G.C. (2019). Patient satisfaction on the utilization of traditional and complementary medicine services at public hospitals in Malaysia. Complement Ther Med.

[56] Fernandez R.C., Marino J.L., Varcoe T.J., Davis S., Moran L.J., Rumbold A.R. (2016). Fixed or rotating night shift work undertaken by women: implications for fertility and miscarriage. Semin Reprod Med.

[57] Ko G.T., Chan J.C., Chan A.W., Wong P.T., Hui S.S., Tong S.D. (2007). Association between sleeping hours, working hours and obesity in Hong Kong Chinese: the 'better health for better Hong Kong' health promotion campaign. Int J Obes (Lond).

[58] Papantoniou K., Massa J., Devore E., Munger K.L., Chitnis T., Ascherio A. (2019). Rotating night shift work and risk of multiple sclerosis in the Nurses' Health Studies. Occup Environ Med.

[59] Weintraub W.S., Lüscher T.F., Pocock S. (2015). The perils of surrogate endpoints. Eur Heart J.

[60] Ciani O., Buyse M., Drummond M., Rasi G., Saad E.D., Taylor R.S. (2017). Time to review the role of surrogate end points in health policy: state of the art and the way forward. Value Health.

